# Prevalence of single umbilical artery, clinical outcomes and its risk factors: A cross-sectional study

**DOI:** 10.18502/ijrm.v19i5.9253

**Published:** 2021-06-23

**Authors:** Homeira Vafaei, Khatoon Rafeei, Maryam Dalili, Nasrin Asadi, Nosaibe Seirfar, Mojgan Akbarzadeh-Jahromi

**Affiliations:** ^1^Maternal-Fetal Medicine Research Center, Shiraz University of Medical Sciences, Shiraz, Iran.; ^2^Obstetrics and Gynecology Department, School of Medicine, Shiraz University of Medical Sciences, Shiraz, Iran.; ^3^Clinical Research Unit, Afzalipour Hospital, Kerman University of Medical Sciences, Kerman, Iran.; ^4^Pathology Department, Jiroft University of Medical Science, Jiroft, Iran.; ^5^Pathology Department, School of Medicine, Shiraz University of Medical Sciences, Shiraz, Iran.

**Keywords:** Umbilical cord, Single umbilical artery, Pregnancy outcome, Congenital abnormalities.

## Abstract

**Background:**

Single umbilical artery (SUA) is found in 0.5–6% of all pregnancies worldwide. Although the association of SUA with some congenital malformations is mainly accepted, its effect on pregnancy/neonatal outcomes is still controversial.

**Objective:**

This is the first study aimed to approximate the SUA prevalence in southern part of Iran. SUA epidemiologic features accompanied by some of its effects on pregnancy/neonatal outcomes are investigated as well.

**Materials and Methods:**

In this cross-sectional study, data from two referral centers in Southern Iran were analyzed. In total, 1,469 pregnancies, fetuses, and neonates were examined for epidemiological features associated with SUA. SUA was confirmed by pathological examination, while congenital anomalies were diagnosed by clinical, ultrasound, and echocardiographical examinations. Data on pregnancy outcome were recorded based on the patients' medical records.

**Results:**

The prevalence of SUA was 3.47% (95% CI: 2.6–4.6%). Fetal anomalies including renal, cardiac, and other congenital anomalies, intrauterine fetal death, early neonatal death, low birth weight, low placental weight, and preterm birth were significantly higher in the SUA group (OR = 68.02, 31.04, 16.03, 3.85, 11.31, 3.22, 2.70, and 2.47, respectively). However, the maternal multiparity was lower in the SUA group (OR = 0.65; 95% CI: 0.44–0.98).

**Conclusion:**

A significant association was observed between SUA and increased risk of intrauterine fetal death and early neonatal death, as well as low birth weight and preterm birth. Obstetrical history of the mother like parity was identified as an important predictor of SUA. Further investigations are suggested on risk stratification of neonates in this regard.

## 1. Introduction

The umbilical cord is the carrier of fetal vein and arteries and any abnormalities in its appearance, composition, location, size, and placental and fetal attachment is associated with fetal death and congenital anomalies (1, 2). Normally, the umbilical cord consists of one vein and two arteries, but the primary agenesis, secondary atresia, or persistent allantoic artery of the body stalk may result in the absence of one umbilical artery, known as single umbilical artery (SUA). Different rates of SUA have been reported for its prevalence, which may vary according to the diagnostic method used and gestational age assessed, as the highest prevalence is reported in abortus and autopsies (0.34–7%) and the lowest in live-born neonates (0.2–1.5%) (3, 4). Correspondingly, the assessment of fetuses by ultrasound at 11–14 wk of gestation resulted in an incidence rate of 5.9% (5). Also, a higher prevalence (4.6–9.8%) is reported in twin pregnancies compared with singleton gestations (6).

SUA can be an isolated finding and is not considered teratogenic alone; nevertheless, previous studies reported its association with congenital anomalies or chromosomal abnormalities (4, 5, 7), and have the potential to increase the odds of neonatal intensive care unit (NICU) admission and mortality rate (8). It is estimated that about 30–60% of cases with SUA are concomitant with congenital abnormalities, like cardiac and genitourinary abnormalities, skeletal or gastrointestinal malformations (3, 9, 10), or chromosomal abnormalities, such as trisomy 13, 18, 21, and triploidy (10). Furthermore, some suggest that the associated comorbidities of SUA result in an increased risk of intrauterine fetal growth restriction (IUGR), polyhydramnios/oligohydramnios, placental abruption, placenta previa, cord prolapse, low Apgar scores, and perinatal mortality (11), while isolated SUA is not associated with increased risk of chromosomal abnormalities (12), adverse perinatal or long-term neurodevelopmental outcomes (13, 14).

As each study has considered SUA in different target populations, including abortuses and autopsies, ultrasound examination, or umbilical cord pathology in fetuses born term or preterm, alive or dead, each have reported a different incidence rate, different rates of isolated SUA, associated comorbidities, or negative perinatal outcomes (9–15). Therefore, this area remains to be further explored in future studies. No previous study addressing the prevalence of SUA and its relationship with pregnancy outcomes in Iran has been published yet and thus the predictors and pathophysiology of SUA are still unknown to us.

The present study aimed to estimate the prevalence of SUA, investigate related epidemiological information, and assess its effects on pregnancy/neonate outcomes in a selected population) in a tertiary referral care center) in Southern Iran. Understanding the predictors of SUA may help us to diagnose high-risk pregnancies earlier and schedule a better prenatal care plan for them, thereby reducing the potential complications.

## 2. Materials and Methods

### Study design

In this cross-sectional study, all consecutive pregnant women with gestational ages over 15 wk referred to two main referral centers (Hafez and Zeynabyie Hospitals affiliated to the Shiraz University of Medical Science, Shiraz, Iran) in southern Iran between October 2012 and October 2013 were recruited. The eligibility criterion for the study was pregnancy over 15 wk of gestational age.

A study checklist, comprising three sections, was designed for the study. In the first section, the demographic characteristics of mothers, including mothers' age, gravidity, and parity, and the gestational age at the time of delivery were recorded. In the second section, possible risk factors related to pregnancy including maternal medical conditions during pregnancy (such as gestational diabetes mellitus, chronic hypertension, preeclampsia, and epilepsy), other maternal diseases such as asthma and anemia, and use of medications was recorded. In the third section, neonatal characteristics, including gestational age at delivery, type of delivery, indications for each type of delivery, infants' sex, birth weight, the first and fifth minutes' Apgar scores, NICU admissions, and presence of significant anomalies were recorded. Renal anomalies were detected by postpartum ultrasound examination and cardiac anomalies through echocardiography.

Preterm birth was considered as childbirth at < 37 wk of gestation and low birth weight (LBW) as birth weight < 2500 gr. All data were recorded from the patients' medical records by the researcher and the missing data were taken from patients during their hospital admission.

After the delivery, the placenta and umbilical cord were sent to the pathology unit of Hazrat Zeinab Hospital in formalin containers and examined within 24 hr after delivery. All samples were examined microscopically by a pathologist who was blind to the outcome and procedure of pregnancy.

### Ethical considerations

The study protocol was approved by the Ethics Committee of Shiraz University of Medical Sciences, Shiraz, Iran (Ethics code: IR.SUMS.REC.1393.4814) and all participants were informed about the study objectives and had signed a written informed consent for participation into the study.

### Statistical analysis

After entering the collected data into the computer, the outliers and missing data were cleared. Data with < 7% missing data were included into the study. After descriptive results were reported, a comparison between the groups was performed using Chi-square and Student's *t* tests. Next, the results were reported by bivariate analysis, and multivariate analyses were defined based on the researchers' hypotheses.

Multivariate models were formed by logistic regression. The confounders were selected in each model, based on biological science. Then, backward elimination was used based on P-value < 0.3 (in bivariate analysis) for selection of confounders in each model. Two-sided P-value < 0.05 was considered as statistically significant. Data were analyzed using the SPSS software for Windows, version 16.0 (Released 2007. Chicago: SPSS Inc.).

## 3. Results

Of the 1,469 pregnant women participating in this study, 51 (3.47%; 95% CI: 2.6-4.6%) had SUA and 57% of them had a female factor. Table I shows the demographic characteristics of the participants.

### Effects of SUA on pregnancy outcomes

The results of 51 SUA and 1,418 double umbilical artery (DUA) pregnancies are shown in Table II. An intrauterine fetal death (IUFD), early neonatal death, preterm birth, low birth weight (LBW), low placental weight, any type of congenital anomalies, cardiac and renal anomalies were significantly higher in the SUA group compared to the DUA group (p < 0.05). However, parity was shown to be a protective factor against SUA (Table II).

IUFD, early neonatal death, preterm birth, LBW, low placenta weight, renal anomaly, cardiac anomaly, any congenital anomalies, C/S, oligohydramnios, and polyhydramnios were higher in SUA group (p < 0.05); however, IUGR and multi-parity did not differ (p > 0.05). Adjusting on GA, no dramatic changes were seen in the significance of the results.

**Table 1:** Maternal, fetal, and neonatal demographic features of all participants, SUA group, and normal umbilical cord group


			**SUA group (n = 51)**	**DUC group (n = 1418)**	**P-value**
**Maternal features**					
	Maternal age (yr), (mean ± SD)*		28 ± 5.56	28.18 ± 6.05	0.80
	Gestational age (wk), (mean ± SD)*		33.63 ± 7.38	36.84 ± 4.96	0.003
	Gravidity (null)${}^{1}$, (%)**		14 (26.9)	410 (28.9)	0.75
	Parity (multi)${}^{2}$, (%)**		14 (26.9)	436 (30.7)	0.55
	Cardiac failure, (%)**		1 (1.9)	2 (0.1)	0.005
	Asthma, (%)**		2 (3.8)	2 (0.1)	< 0.001
	Diabetes mellitus, (%)**		1 (1.9)	32 (2.3)	0.87
	Anemia, (%)**		2 (3.8)	7 (0.5)	0.002
	Gestational hypertension, (%)**		2 (3.8)	24 (1.7)	0.25
	Hypothyroid, (%) **		0 (0.0)	13 (0.9)	0.50
	Rout of termination (cesarean), (%)**		34 (66.7)	749 (52.8)	0.052
**Fetal features**					
	Placenta weight (gr), (mean ± SD)*		409.73 ± 174.71	489.96 ± 204.50	0.018
	Low placenta weight (%)**				
	**Yes**	11 (21.6)	172 (12.1)	0.01
	**No**	40 (78.4)	1246 (87.9)	
	IUGR, (%)**		2 (3.9)	49 (3.5)	0.86
	Oligohydramnios, (%)**		3 (5.8)	18 (1.3)	0.007
	Polyhydramnios, (%)**		1 (1.9)	4 (0.3)	0.046
	IUFD, (%)**		6 (11.5)	27 (1.9)	< 0.001
**Neonatal features**					
	Preterm, (%)**		24 (46.2)	359 (25.3)	0.001
	Cardiac anomaly, (%)**		11 (21.6)	13 (0.9)	< 0.001
	Renal anomaly, (%)**		7 (13.7)	3 (0.2)	< 0.001
	Other anomalies, (%)**		12 (23.5)	25 (1.8)	< 0.001
	Early neonatal death, (%)**		8 (15.7)	9 (0.6)	< 0.001
	Gender (%)**				
	**Female**	30 (58.8)	676 (47.67)	0.11
	**Male**	21 (41.2)	742 (52.33)	
	Birth weight (gr), (mean ± SD)*		2172.25 ± 1087.87	2871.14 ± 872.02	< 0.001
	LBW, (%)**				
	**Yes**	23 (45.09)	295 (20.8)	<0.001
	**No**	27 (54.91)	1123 (79.2)	
	1${}^{\mathrm{𝐬𝐭}}$ min Apgar score, (mean ± SD)*		6.14 ± 3.43	8.06 ± 2.17	< 0.001
	5${}^{\mathrm{𝐭𝐡}}$ min Apgar score, (mean ± SD)*		7.11 ± 3.92	9.18 ± 2.25	< 0.001
	NICU admission, (%)**		34 (66.7)	17 (33.3)	< 0.001
*Student's *t* test was used, **Chi-square test was used, ${}^{1}$Null gravid is gravid = 1, IUGR: Intrauterine growth retardation, IUFD: Intrauterine fetal death, DUC: Double umbilical cord, NICU: Neonatal intensive care unit, SUA: Single umbilical artery, LBW: Low birth weight (birth weight < 2500 gr), low placenta weight: placenta weight < 10${}^{\mathrm{th}}$ percentile					

**Table 2:** Comparison of pregnancy and neonatal outcomes in two study groups


	**Univariate logistic results**	**Multivariate logistic results**
**Features**	**OR (95% CI)**	**OR (95% CI)**
**IUFD**	3.85 (1.28–11.61)	3.2 (1.1–9.48)
**Early neonatal death**	11.31 (2.83–45.26)	10.24 (2.4–42.34)
**Preterm birth**	2.47 (1.40–3.40)	2.01 (1.1–2.96)
**LBW**	3.22 (1.78–5.83)	2.4 (1.12–5.15)
**Low placenta weight(< 10${}^{\mathrm{𝐭𝐡}}$ percentile)**	2.70 (1.13–6.44)	1.17 (0.44–3.05)
**Renal anomaly**	68.02 (16.38–282.35)	67.83 (16.36–276.657)
**Cardiac anomaly**	31.04 (12.05–79.94)	25.59 (10.69–61.22)
**Any congenital anomalies**	16.03 (7.21–34.07)	12.4 (5.23–29.36)
**IUGR**	0.99 (0.23–4.32)	1.01 (0.2–4.3)
**C/S**	1.72 (0.95–3.13)	1.5 (0.64–2.45)
**Oligohydramnios**	4.05 (1.10–14.90)	4.02 (1.01–13.8)
**Polyhydramnios**	8 (1.05–73.2)	6.27 (3.42–11.47)
**Multi-parity**	0.65 (0.44–0.98)	0.73 (0.3–1.4)
IUFD: Intrauterine fetal death, IUGR: Intrauterine growth retardation, OR: Odds ratio, CI: Confidence interval, SUA: Single umbilical artery, LBW: Low birth weight (birth weight < 2500 gr), C/S: Cesarean section. Features were compared using multivariate logistic regression models. Multi-parity: parity ≥ 2		

## 4. Discussion 

The present study investigated 1,469 neonates born alive, among whom 51 (3.47%) had SUA with a female-to-male ratio of 1.32:1. Congenital anomaly was observed in 23.5% newborns of SUA group. There were 27 IUFD cases and 10 early neonatal deaths, resulting in a total mortality rate of 72.5% for neonates born with SUA. According to the statistical analysis, history of no live birth in the mother was identified as an important risk factor for SUA.

Although SUA is considered as the most common vessel abnormality of umbilical cord, the prevalence of SUA reported in live births in previous studies, which have examined the umbilical cord after delivery (0.2–1.5%) (16, 17), is lower than that of the present study (3.5%). Studies investigating > 4000 umbilical cords have also reported a general prevalence of < 1% (15), which is again lower than the present study. This higher prevalence may be related to the reporting of the results of a selected population, who were referred to the studied center for a specific condition (18); for instance, more high-risk pregnancies might have been included in our study due to the referral nature of our center. Another difference regarding the prevalence rates of SUA reported may be related to the differences in time and method of evaluation (3, 19). Nevertheless, the prevalence of SUA reported in the present study is lower than the studies investigating autopsies, which reported incidence rates as high as 7% (3). Another important factor in this regard is that the prevalence of SUA may be affected by the race/ethnicity of the studied population (16). The prevalence rates reported in the studies in neighboring countries, like Turkey (1.04%) (20) and Saudi Arabia (0.63%) (21), conducted on the general population, are lower than that reported in our study confirmed with histology examination.

As reported in the present study, SUA resulted in a 16-fold increase in the odds of congenital anomalies, while 76.5% of neonates with SUA had no congenital anomalies, which is consistent with previous reports indicating isolated SUA in about 65% of cases (22). In addition, among the cases with congenital anomalies in the present study (23.5%), cardiac and renal anomalies were detected in eight and nine cases (19.04% and 21.42%), respectively. These results are in line with previous studies reporting genitourinary and cardiovascular anomalies as the most common congenital anomalies in cases with SUA, while gastrointestinal anomalies are considered the least frequent ones (23–25). Further, the results of a meta-analysis also suggested that examination for cardiac malformations is necessary, even in cases with isolated SUA (25). The study by De Figueiredo and colleagues reported cardiac anomalies in 6.5% of cases with SUA, detected by second-trimester ultrasound examination (25), which is lower than the present study. Similarly, Murphy-Kaulbeck and colleagues reported genitourinary anomalies in 6.48% and cardiovascular anomalies in 6.25% of their cases (23), which is lower than the anomaly rates reported in our study. This difference could be because they have reported the anomalies in chromosomally normal fetuses and neonates, while we evaluated all pregnancies over 15 wk of gestation who gave birth at this center, which could also include chromosomal abnormalities Prucka and colleagues have similarly reported that only 4 of the 22 cases with cardiac anomaly were isolated in their study and emphasized on the importance of cardiac assessment, even in cases with isolated SUA (19). A wide range of cardiac anomalies has been reported in similar studies (23–26), which indicates the important association of SUA with cardiac anomalies. In the study by Hua and colleagues, renal anomalies were present in 4.8% of cases with SUA among 64,047 pregnancies studied (16), which, though lower than that of the present study, emphasizes on the significance of renal anomalies in these neonates, although, the pathogenesis and etiology of such association remains unclear.

Another important finding in the present study was the high rate of IUFD (52.94%) and significantly lower mean gestational age in neonates with SUA, compared with those without, resulting in the adjusted effect size of 3.85 (95% CI: 1.28–11.61) and 11.31 (95% CI: 2.83–45.26) for IUFD and early neonatal death. Similar to these results, other studies have also reported an increased risk of IUFD and preterm birth in neonates with SUA (23, 27), although the exact nature and mechanism of this association is not clearly understood. It is supposed that an increase in resistance to placental blood flow may result in a decrease in the delivery of oxygen and other nutrients, which predispose the fetus to IUFD (28).

Also, the results of the present study indicated a low birth weight for neonates with SUA, which is consistent with the results of previous studies, indicating an increase in the odds of LBW or small-for-gestational age and preterm birth in neonates or fetuses with SUA (8, 29, 30). According to the results of the present study, SUA resulted in a high mortality rate of neonates, which is in line with previous studies (8, 31, 32), indicating SUA as an independent risk factor for adverse perinatal outcome in term neonates (33, 34), which along with the results of the present study emphasizes the importance of this issue.

In the present study, there were only two cases of IUGR in patients with SUA and the risk of IUGR was not different in neonates with or without SUA, which is contrary to the results of previous studies. They reported an increased risk of IUGR and preterm birth in neonates with SUA. The hypothesized mechanism of this association may be similar to the underlying mechanism for IUFD in these neonates (23, 27). However, similar to the results of the present study, some researchers have also detected no association between IUGR and SUA (14, 27). According to the controversial results on this issue, more extensive studies are required to elucidate the exact association between SUA and IUGR.

As discussed earlier, several studies have investigated the prevalence and adverse outcomes of SUA, nevertheless, few have investigated its risk factors and predictors. The present study showed the protective effect of multiparity on SUA. Predicting SUA, irrespective of its pathophysiology, can help us detect the high-risk population and prevent the potential complications by close and better prenatal care. Meanwhile, like any other study, it too had some limitations. Firstly, the cross-sectional nature of the study limited the assumption of any causal relationship between the factors associated. Secondly, our study was conducted at two tertiary perinatal centers, and referral center bias may limit the ability of our results to be generalized to community-based patients.

## 5. Conclusion

As indicated in the present study, a significant increase in the odds of IUFD, preterm birth, and LBW was observed in neonates with SUA. These findings could be used in counseling of women whose pregnancies are complicated by SUA. In future, appropriate antenatal surveillance could be offered to such population, which will theoretically reduce the adverse perinatal outcome of the SUA group. Evaluating the obstetrical history of the mother, such as parity, were identified as important predictors of SUA and are suggested to be studied for risk stratification of neonates.

##  Conflict of Interest 

The authors declare that there is no conflict of interest regarding the publication of this paper.
